# Prevalence and clinical characteristics of patients with Advanced Chronic Illness and Palliative Care needs, identified with the NECPAL CCOMS-ICO© Tool at a Tertiary Care Hospital

**DOI:** 10.1186/s12904-022-01101-4

**Published:** 2022-11-28

**Authors:** Agnès Calsina-Berna, Jordi Amblàs Novellas, Jesús González-Barboteo, Ignasi Bardés Robles, Elba Beas Alba, Marisa Martínez-Muñoz, Rafael Madariaga Sánchez, Xavier Gómez Batiste Alentorn

**Affiliations:** 1 Palliative Care Service. Research and knowledge group in palliative care of Catalan Institute of Oncology (GRICOPAL), Institut Català d’Oncologia-Badalona, Badalona, Spain; 2 Central Catalonia Chronicity Research Group, Chair of Palliative Care, Faculty of Medicine, University of Vic-Central University of Catalonia, Vic, Spain; 3 Palliative Care Service. Research and knowledge group in palliative care of Catalan Institute of Oncology (GRICOPAL), Institut Català d’Oncologia- L’Hospitalet. Hospitalet de Llobregat, Barcelona, Spain; 4 Emergency Department, Hospital Universitari de Bellvitge, Barcelona, Spain; 5 Chair of Palliative Care, Faculty of Medicine, The Qualy Observatory, WHO Collaborating Center for Palliative Care Public Health Programs, University of Vic/Central Catalonia, Catalan Institute of Oncology, Barcelona, Spain; 6 Department of Economics, University of Vic-Central University of Catalonia, Barcelona, Spain

**Keywords:** Terminal care, Palliative care, Chronic conditions, Hospital care

## Abstract

**Background:**

The identification of patients with advanced chronic conditions and palliative care needs is essential since their care represents one of the main challenges for public health systems. The study aimed to determine the prevalence and characteristics of inpatients with palliative care needs in different services of a tertiary care hospital using the NECPAL CCOMS-ICO© tool.

**Methods:**

A descriptive, cross-sectional cohort study was conducted in a tertiary hospital.

The NECPAL tool identifies patients who require palliative care. Any patient with the Surprise Question with the answer “NO” and at least another question of the tool with a positive answer is considered a positive identification.

Patients were classified as Non-NECPAL, NECPAL I-II, and NECPAL III, depending on the NECPAL tool criteria they met. The presence of physical symptoms, emotional distress, and social risk factors was assessed.

**Results:**

Of the 602 inpatients, 236 (39.2%) were enrolled. Of them, 34 (14.4%) non-NECPAL, 202 (85.6%) NECPAL+ [105 (44.5%) NECPAL I-II, and 97 (41.1%) NECPAL III]. Physical symptom burden was high (pain intensity ≥ 1 in 68.3% of patients; tiredness ≥ 1 in 83.5%; somnolence ≥ 1 in 50.6%; dyspnea ≥ 1 in 37.9%; anorexia ≥ 1 in 59.5%). 64.1% had emotional distress, and 83.6% had social risk factors. The NECPAL-III group contained a higher percentage of cancer patients, higher demand for palliative care, and greater need for palliative care (p < 0.001). In 50.8% of cases, no referrals were made to psychology, social work, or hospital palliative and supportive care teams. The three services with the higher number of patients with palliative care needs were: Palliative Care Unit (100%), Oncology (54.54%), and Emergency Short-stay Unit (54.16%).

**Conclusion:**

A high percentage of patients admitted to tertiary care hospitals presented palliative care needs, with multiple unmet physical, emotional, and social needs. Less than 50% are referred to specialized care teams, such as hospital palliative and supportive care teams.

**Supplementary Information:**

The online version contains supplementary material available at 10.1186/s12904-022-01101-4.

## Background

Currently, care for patients with advanced chronic conditions represents one of the main challenges for public health systems. Identifying patients with palliative care needs is fundamental with relative independence of prognosis and level of complexity. This identification might determine the need to introduce a palliative approach regardless of the service or the patient’s setting.

Many patients experience a physical and nutritional decline during their illness, with an emotional, social, and spiritual impact and frequent needs and demands crises. Additionally, these illnesses can lead to ethical dilemmas for healthcare professionals regarding the heavy use of resources and considerable suffering for patients and family members [1]. Such patients can receive care in most health service settings, including acute care hospitals [2], intermediate care facilities, emergency services, community [3], and nursing homes. In patients with advanced chronic illnesses, 69-82% will require palliative care before death [4].

Inpatients tend to present numerous burdensome symptoms that are difficult to manage as far as acute care hospitals are concerned. As the disease progresses, hospital admissions increase regardless of where patients die. In Spain, 52% of patients with non-sudden deaths are admitted at least once in the last three months of life [5].

To better care for patients with palliative care needs, there must be a consensus among all health services to identify hospitalized patients with an advanced stage of their diseases. As there is no clear definition, this identification becomes a challenge, and most remain at the hospital without receiving this special care [6, 7]. Therefore, the lack of understanding of when a patient requires palliative care [8] can lead to delays. Thus, suffering for patients and their environment [7, 9].

In recent years there have been innovations in palliative care. One of the “Conceptual Innovations” has been the consideration that patients should not only be treated at the final stages of their disease but before. For this reason, the concepts of “First Transition” and “Second Transition” palliative care have been developed [10].

Several initiatives have been developed to create tools to identify patients with palliative care needs. For example, the Gold Standards Framework [11] in England, the SPICT in Scotland, and a similar tool in Spain, the “NECPAL (NECesidades PALiativas, palliative care needs) tool” (from now on, NECPAL), were created. The Gold Standards Framework - Proactive Identification Guidance (GSF-PIG) has been used to determine the prevalence of patients with palliative care needs in hospital settings in other countries, with reports ranging from 20 to 36% of inpatients [12–15]. Currently, the GSF-PIG is used in the UK to identify such patients in the hospital[16].

The NECPAL is a validated tool used in primary care to identify advanced chronic patients with palliative care needs that can be used in any area of the health system. It introduces improvements in the quality of palliative care in all services. This instrument assessed the prevalence of individuals with palliative care needs in the general population (1.5%) [3]. It has also been employed as part of a population study in a secondary hospital [3]. However, NECPAL has not been used in an entirely tertiary care hospital. Thus, the prevalence and clinical characteristics of NECPAL + patients in tertiary hospitals are unknown.

The study’s primary aim was to identify advanced chronic patients with palliative care needs (NECPAL+) in tertiary hospital services through the NECPAL tool. A secondary objective was to determine their symptoms and whether there were significant differences in the clinical characteristics of patients depending on the number of criteria met with the NECPAL tool. Based on the number of criteria, patients could be classified with different degrees of severity.

## Materials and methods

### Study design

It was a descriptive, cross-sectional cohort study conducted in two public hospitals, the Bellvitge University Hospital (HUB), with 759 beds, which is a tertiary hospital covering a population area of more than 2 million inhabitants, and the Duran i Reynals Hospital (DIR), with 97 beds, the associated HUB cancer center. All the hospital areas were assessed except Emergency departments and other services without hospital beds.

A cross-sectional cut was performed at each hospital department with a different cut-off date. Before the fieldwork, each service head of the included services and the corresponding nursing supervisors were invited to participate in the study. Individual meetings were held with each service to explain it, offer clarifications, and determine the date for the cross-sectional data collection.

The study was conducted following the principles outlined in the revised version of the Declaration of Helsinki and Good Clinical Practices (GCPs) and was approved by the Bellvitge University Hospital Ethics Committee (Reference PR069/12). Signed informed consent was obtained from all patients who answered the questionnaires.

### Patient recruitment and data sources

The inclusion criteria for the study were individuals ≥ 18 years of age with a minimum hospital admission of 24 h and with any of the eight selected chronic illnesses (cancer, chronic obstructive pulmonary disease, chronic heart disease, chronic neurological disease (either vascular or degenerative), serious chronic liver disease, serious chronic renal disease, dementia, and advanced frailty), as well as any other advanced conditions.

The inclusion process was done as follows: (1) The inpatients of the cross-sectional day were obtained through a computerized list, and the clinical and demographic data were obtained from their respective medical records. (2) The doctor and nurse responsible for each patient determined which patients were particularly affected by their chronic disease. This condition was noted as the “main diagnosis.” The data manager of the Research Unit collected the variables in a Clinical Research Document (CRD) from the medical records, except for those who needed patients’ answers. The responsible physician or nurse was consulted if data were incomplete or unclear in the medical records. (3) Patients enrolled were invited to answer scales: Edmonton Symptom Assessment Scale (ESAS) and Detection of Emotional Distress (DED), and information related to social risk factors -see variables.

The study sample size was achieved when all services were included.

### Variables analyzed

After patient enrollment, the NECPAL CCOMS-ICO^©^ tool was administered [3]. This tool includes a multifactorial, non-dichotomous assessment process that combines the Surprise Question (SQ) “*Would you be surprised if this patient died in the next year*?” with the presence of 3 groups of criteria or variables: (Point 2) choice/request or need of palliative care approach; (Point 3) general clinical indicators of severity and progression, including comorbidity and resource use; and (Point 4) specific clinical indications of severity and progression per diseases. Patients considered positive for NECPAL are SQ + patients who also fulfill at least one of the other three tool criteria.

Furthermore, this allows patients to be classified into four categories: (1) Non-NECPAL, when the patient did not meet any of the tool’s criteria; (2) SQ+, when doctors or nurses would not be surprised if this patient died in the next year; (3) NECPAL I-II, when the patient was NECPAL + and met a maximum of two additional criteria; (4) NECPAL III, when the patient was NECPAL + and met three or more of the NECPAL tool criteria.

For all patients included, the variables assessed in addition to NECPAL criteria were: age; sex; main diagnosis; functional status; the presence of cognitive decline with the Pfeiffer test [17]; the presence of comorbidity with the Charlson index [18]; the presence of social risk factors [19]; pharmacological treatments, especially the use of analgesics; previous hospital admissions; and interdepartmental consultations (psychology, social work, or hospital palliative and supportive care).

Patients who agreed with it completed the ESAS scale [20] and the DED scale. The ESAS scale includes questions about symptoms, such as pain, tiredness, nausea, depression, insomnia, and perception of wellbeing. The DED scale assesses the presence of emotional distress. Before answering the ESAS and DED scales, all patients completed the Pfeiffer questionnaire [17], which assesses the presence of cognitive impairment. Those with cognitive impairment did not answer the ESAS and DED scales. If patients had cognitive impairment, relatives could answer questions related to social risk factors.

### Statistical analysis

Quantitative variables were described as the mean and standard deviation (SD), whereas categorical variables were described as frequency and, or percentage. To compare quantitative variables, we used the Student t-test or ANOVAs when the variable had a normal distribution and the Kruskal-Wallis test for non-normal distributions.

## Results

### Patient characteristics

Twenty-seven departments were evaluated, with 602 inpatients, 366 (60.8%) did not meet inclusion criteria, and 236 (39.2%) were enrolled. The median age was 68.2 years (SD 14.72), and 143 (61%) were women. No significant differences were shown in gender between included and non-included patients. On the contrary, significant age differences were observed, with the mean age of the included patients being 68.20 years (SD 14.72) and of not included patients, 60.83 years (SD 15.72) (*p* = < 0.001). No significant differences in age and sex were found between the patients who signed the informed consent and those who did not: 67.96 years (SD 15.1) vs. 68.49 years (SD 14.32), and 75 men (58.59%) vs. 69 men (63.88%), respectively.

### Prevalence results

Of the 602 patients admitted to the hospital, 236 (39.2%) were included in the study as affected by chronic conditions; 202 (33.55%) were NECPAL + [105 (17.44%) were NECPAL I-II, and 97 (16.11%) were NECPAL III]. Supplemental Table S1 shows the distribution of admitted patients, those included and those identified with the NECPAL CCOMS-ICO© instrument for each service. The three services with more patients with palliative care needs were: Palliative Care Unit 100%, Oncology 54.54%, and Emergency Department Short Stay Unit 54.16%.

**Fig. 1:**
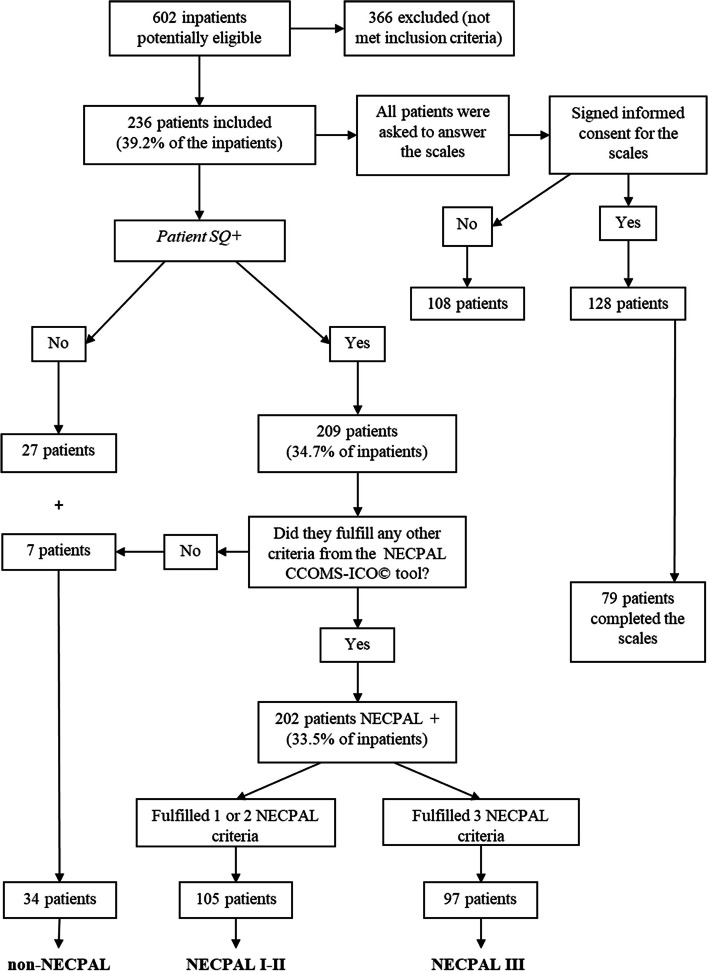
Study flowchart

Figure 1 shows the study patients’ flowchart and NECPAL groups. Of the patients included, 79 without cognitive impairment answered the ESAS and DED scales.

### Physical symptoms according to ESAS

Supplemental Table S2 (supplemental material) lists the prevalence and intensity of patients’ symptoms by NECPAL group, according to the ESAS Scale. Differences among the three categories (non-NECPAL, NECPAL I-II, and NECPAL III) were analyzed. As mentioned, the ESAS was completed by 79 patients; of these, 36 patients (45.6%) presented pain of more than two weeks’ duration. 50.63% of patients had pain with a severity of 4 or more on the Visual Analog Scale, with a mean intensity of pain of 3.51 (SD 3.16) and a median of 4 (range 0–10).

### Other variables assessed

Slightly less than one-third (31.3%) had some type of cancer (by category: 0% non-NECPAL; 26% NECPAL I-II; 48.5% NECPAL III) (p < 0.001).

The descriptive analysis of the variables related to the NECPAL tool is shown in Table 1.

**Table 1:** Patient characteristics according to NECPAL tool variables

Variables, n (%)	Non-NECPAL *n* = 34	NECPAL I-II *n* = 105	NECPAL III *n* = 97	TOTAL *n* = 236	P value
Choice/request for PC	0 (0%)	14 (13.3%)	38 (39.2%)	52 (22%)	< 0.001
Need for PC	5 (14.7%)	51 (48.6%)	89 (91.8%)	145 (61.4%)	< 0.001
Weight loss > 10%	4 (20%)	34 (50.7%)	45 (60%)	83 (51.2%)	0.006
Barthel Index < 20	5 (17.2%)	27 (29.3%)	47 (50.5%)	79 (36.9%)	0.001
Loss ≥ 2 ADL	6 (22.2%)	44 (58.7%)	61 (73.5%)	111 (60%)	< 0.001
Functional decline*	5 (20%)	55 (67.9%)	71 (81.6%)	131 (67.9%)	< 0.001
Ulcers	0 (0%)	12 (11.7%)	9 (9.4%)	21 (9.1%)	ns
Recurrent infections	7 (24.1%)	41 (43.6%)	40 (44%)	88 (41.1%)	ns
Delirium	4 (11.8%)	21 (22.6%)	26 (29.9%)	51 (23.8%)	ns
Persistent dysphagia	0 (0%)	13 (13.5%)	23 (26.4%)	36 (17%)	0.002
Falls > 2	3 (17.6%)	14 (20.6%)	7 (10.8%)	24 (16%)	ns
≥ 2 admissions‡	10 (35.7%)	28 (31.5%)	41 (48.8%)	79 (39.3%)	ns
Complex care^§^	6 (18.2%)	45 (43.3%)	47 (49%)	98 (42.1%)	0.008
Charlson Index ≥ 2	23 (67.6%)	74 (70.5%)	71 (73.2%)	168 (71.2%)	ns
Albumin ≥ 2.5 g/dl	2 (7.7%)	15 (17.2%)	16 (19.8%)	33 (17%)	ns

Need for PC: * Clinicians were asked: “Do you consider that this patient requires palliative treatment at this moment?

Functional decline: clinical perception of functional decline.

† Persistent pressure ulcers (stage III-IV).

‡ ≥ 2 unplanned emergency hospital or skilled nursing facilities admissions due to chronic disease in the last year.

^§^Need of complex/intense continuing care, either at an institution or at home.

**Abbreviations**: *n*, number of patients, *%* percentage of patients, *PC* palliative care, *ADL* activities of daily living, *ns* not significant.

**Emotional Distress through the DED Scale**: Based on the DED, 64.1% of patients presented emotional distress, with a mean on the DED scale of 10.28 (5.48) and a median of 10 (6.75-14).

**Comorbidity with the Charlson Index**: No statistically significant differences among the different groups were seen on the Charlson index. Mean scores were as follows: non-NECPAL group (mean score = 2); NECPAL I-II (mean, 3); and NECPAL III (mean score, 3).

**Social Risk Factors**: 128 patients (54.23%) or relatives could answer the questions related to social risk factors. Most patients, 197 (83.60%), had at least one social risk factor: 6.66% of the patients were illiterate; 66.66% had completed primary education, and only 7.50% had completed university studies; 13.29% of the patients lived alone; 12.88% had a main caregiver older than 80 years old; in 21.88% of cases, there were other members in the environment that needed care; 37.80% commented that they would need help from an external caregiver; 32.82% considered the health status of their caregiver as fair/bad or very bad; 37.60% would need help.

**Pharmacological Treatments**: Table 2 shows the medications, especially the use of analgesics. Of patients with pain (Supplemental Table S2), 19.50% were receiving opioids: 2.95% of non-NECPAL patients, 16.20% of NECPAL I-II patients, and 28.90% of NECPAL III (p = 0.002); with a mean of 9.29 drugs per patient.

**Table 2:** Medical prescriptions in place on the day of study inclusion and mean number of medications per patient

	Non-NECPAL *n* = 34	NECPAL I-II *n* = 105	NECPAL III *n* = 97	TOTAL *n* = 236	P value
**Analgesics**, n (%)					
Analgesic	19 (55.9%)	64 (62.2%)	70 (73.7%)	153 (65.9%)	ns
Metamizole	5 (14.5%)	13 (12.7%)	13 (13.7%)	31 (13.4%)	ns
NSAIDS	1 (3%)	13 (12.7%)	11 (11.6%)	25 (10.8%)	ns
Paracetamol	15 (44.2%)	42 (40.8%)	38 (40%)	95 (40.9%)	ns
Codeine	0 (0%)	1 (1%)	0 (0%)	1 (0.5%)	ns
Tramadol	2 (5.9%)	4 (3.9%)	3 (3.2%)	9 (3.9%)	ns
Morphine	0 (0%)	3 (2.9%)	7 (7.4%)	10 (4.3%)	ns
Fentanyl	1 (3%)	13 (12.7%)	12 (12.8%)	27 (11.7%)	ns
Methadone	0 (0%)	0 (0%)	6 (6.4%)	6 (2.6%)	0.012
Buprenorphine	0 (0%)	1 (1%)	2 (2.2%)	4 (1.8%)	ns
**Other drugs**, n (%)					
Neuroleptics	4 (11.8%)	11 (10.7%)	21 (22.2%)	36 (15.5%)	ns
Benzodiazepines	17 (50%)	32 (31.1%)	38 (40%)	87 (37.5%)	ns
Antidepressive	6 (11.2%)	19 (18.5%)	15 (%)	40 (17.4%)	ns
AC therapy	6 (17.7%)	8 (7.7%)	7 (7.4%)	20 (8.7%)	ns
Corticoids	12 (36.4%)	30 (29.2%)	32 (33.7%)	74 (32%)	ns
GP	30 (88.3%)	85 (82.6%)	76 (80.9%)	191 (82.7%)	ns
Antibiotics	13 (38.3%)	42 (40.8%)	45 (47.4%)	100 (43.1%)	ns
Laxatives	6 (18.2%)	32 (31.1%)	34 (35.8%)	72 (31.2%)	ns
**Number of Medications Overall**					
Mean (SD)	8.76 (2.89)	9.37 (3.19)	9.40 (3.7)	9.29 (3.36)	
Median (P25-P75)	9 (7–10)	9 (7–12)	9 (7–12)	9 (7–12)	ns

**Abbreviations**: *n* number of patients, *%* percentage of patients, *ns* not significant, *AC* anticoagulant, *GP* gastric protectors, *NSAIDS* Non-steroidal anti-inflammatory drugs, *SD* standard deviation, *P25* Percentile 25, *P75* Percentile 75.

** Previous Hospital Admissions and Interdepartmental Consultations**: For 50.80% of patients, no referrals were made to specialized care teams or units (psychology, social work, or hospital palliative and supportive care). Table 3 shows the duration of hospitalization, number of hospital admissions in the last year, and number of stays in the emergency department in the previous year.

**Table 3:** Duration of admission, hospital admissions and stays in the emergency department in the last year

	Non-NECPAL *n* = 34	NECPAL I-II *n* = 105		NECPAL III *n* = 97		TOTAL *n* = 236	P value
**Admissions in the last year**, n (%)							
Hospital	8 (40%)	21 (41.2%)		36 (63.2%)		65 (50.8%)	0.043
Emergency Department	11 (55%)	33 (64.8%)		48 (84.3%)		57 (61.3)	0.015
**Abbreviations**: *n* number of patients, *%* percentage of patients, *SD* standard deviation.							
**Duration of admission** (days)	**Overall** *n* = 236		**Medical speciality** *n* = 166		**Surgical speciality** *n* = 70		**P value**
Median (P25-P75)	22 (11.25-40)		20 (9-35.5)		34 (15–66)		< 0.001
**Abbreviations**: *n* number of patients, *P25* Percentile 25, *P75* Percentile 75.							

## Discussion

The most relevant contribution of the study is the assessment of the prevalence of patients with palliative care needs in a tertiary hospital. The results have shown that a high percentage of patients with palliative care needs (33.55%) could not be identified without the systematic use of a tool. Most of them have symptoms to control and social risk factors.

The use of the NECPAL CCOMS-ICO© instrument is not limited to a specific region or a specific country. In recent years, its use has been widespread in different countries. In 2021, it was validated in Chile by Troncoso et al. [21] and Brazil [22]. This tool was used to identify patients’ conditions and palliative care in five Italian regions [23] and a Portuguese liver unit [24]. A study with the NECPAL tool has also been carried out in a tertiary hospital but only in the Internal Medicine Service, and in a regional hospital assessing all services but with small sample size and without assessing patients’ symptoms [25].

The NECPAL tool is not only a tool for research purposes, but it is a feasible tool that can be used in the clinical setting and whose application does not exceed ten minutes when there is sufficient clinical knowledge of the patient. The NEPCAL tool could be implemented as a routine assessment to better identify patients with palliative care needs and could be completed by the physician in charge of the patient, together with the nurse, during admission of hospitalized patients, at least 24 h after admission entry.

Numerous studies have also been carried out in the hospital environment using other tools, such as GSF-PIG or the Gold Standards Framework to identify individuals with palliative care needs. However, in many of them, patient input was not included [14]. In addition, even when patient input was included, fewer variables were collected compared to our study [12].

The prevalence rates found in the present study are consistent with rates described in other studies conducted in our geographical area and internationally. The prevalence in our study (33.5%) is within this rate; Zertuche et al. [2] found that 25% of inpatients presented were advanced-terminally ill. Studies in other countries using the GSF-PIG to identify patients with PC needs have reported prevalence rates ranging from 19 to 36% [12, 14, 15].

By NECPAL category, we found that the most significant proportion of NECPAL III patients (48.5%) were cancer patients; similarly to Gott et al. [15], who found that 47% of patients met at least one GSF-PIG criterion had cancer. Notably, the NECPAL III patients presented more weight loss and functional decline in the last six months. For this reason, the inpatients most affected by chronic illness are those who have cancer as a chronic condition. In other words, cancer patients not only make up the largest group of NECPAL III patients but are also the most affected by their illness.

Patients with chronic illnesses presented a higher rate of comorbidities [26, 27]. Our findings are consistent with the data reported in other studies conducted in hospital settings, in which Charlson index scores ranged from three to four [28–30].

Based on the ESAS scale, we found no significant differences in physical symptoms among the three NECPAL groups. This finding could be related to our population, which was mainly made up of hospitalized patients admitted due to acute symptoms related to an intercurrent process, which can lead to inadequate symptom control in all groups. Thus, variables related to the patient’s previous deterioration (due to the chronic illness rather than the current intercurrent process) significantly differed among the three NECPAL groups. NECPAL III patients showed more significant deterioration in weight loss, Barthel index < 20, loss of ≥ 2 activities of daily living, functional deterioration, dysphagia, and the need for complex care. Naturally, these patients were most affected by their chronic illness before admission.

Although we did not find any significant differences among the groups in terms of symptom severity and prevalence, symptoms reported through the ESAS scale were highly prevalent in all groups, a fact that is consistent with previous reports in which patients admitted for chronic complex and advanced illnesses present a high burden of symptoms [31].

Moreover, the high percentage of patients with pain is consistent with previous reports, in which 50% of patients present pain [32], which is often undermedicated [33]. Other studies have also reported scant use of opioids, and previous reports have shown that their use in Spain is below the European average [34].

In terms of drug prescriptions at the time of enrollment, patients in the study sample were taking many medications. This fact was comparable to other reports in our region, in which inpatients were taking an average of 7 to 8 drugs each [28, 35, 36]. In this sense, it is important to stress that inappropriate medication can lead to adverse healthcare results, including severe side effects and unnecessary hospitalizations related to polypharmacy [36], associated with high mortality rates [37].

It was not directly assessed whether clinicians followed specific criteria to refer patients for interdepartmental consultations. It is well-documented that interdisciplinary palliative care teams positively affect the course of the illness in patients and families [38, 39] and even survival [40], which means that greater involvement of these teams would be beneficial. Thus, after the identification with the NECPAL tool, NECPAL-positive patients could also be screened with other tools to determine their complexity, such as the PALCOM tool [41]. The PALCOM tool includes socio-demographic and clinical data, symptom burden, functional and cognitive status, psychosocial problems, and existential-ethical dilemmas. Based on this multidimensional evaluation, patients are classified as high, medium, or low palliative complexity, associated with needing basic or specialized palliative care teams. Patients could be referred to specific palliative care teams depending on their complexity, but to do so, they should first be identified with the NECPAL tool.

Finally, we demonstrated significant differences in the clinical characteristics of patients stratified according to disease severity measured by the NECPAL (NECPAL I-II versus NECPAL III).

One of the main strengths of this study is that a single researcher performed data collection. Moreover, this same researcher was responsible for all in-person interviews with patients and healthcare professionals. This approach ensured consistency in data collection.

The study’s limitations include that, although the ESAS and DED scales have only been validated in cancer populations, we applied these tools to the whole sample, which included a sizeable percentage of non-cancer patients. However, given the widespread use of these tools and the lack of scales with similar characteristics for non-cancer populations, we believe thattheir use in this study was justified. Finally, the study was conducted in a single acute care hospital (and its associated cancer center); as a result, it may not be possible to generalize these results to other centers or countries.

In summary, many patients admitted to a tertiary care hospital presented palliative care needs, with multiple physical, emotional distress, and social needs. The availability of a tool like NECPAL would allow the identification of these patients without a subjectivity bias. Although there are significant differences in the clinical characteristics of patients based on disease severity measured by different NECPAL groups, our findings would need to be confirmed by similar studies in different hospitals.

## Supplementary information

Additional file 1: **Supplementary Table S1.** Distribution of admitted patients, those included, those identified with the Surprise Question and those identified with the NECPAL CCOMS-ICO © instrument for each service.

Additional file 2: **Supplementary Table S2.** Prevalence and intensity of symptoms according to the ESAS scale.

## Data Availability

The datasets used during the current study are available from the corresponding author upon reasonable request.

## Abbreviations

CRD: Clinical Research Document
DED: Detection of Emotional Distress
DIR: Duran i Reynals Hospital
EDSSU: Rheumatology, Emergency Department Short Stay Unit
ESAS: Edmonton Symptom Assessment Scale
GAU: Internal Medicine, Geriatric Assessment Unit
GCPs: Declaration of Helsinki and Good Clinical Practices
GSF-PIG: The Gold Standards Framework - Proactive Identification Guidance
HUB: Bellvitge University Hospital
PCU: Palliative Care Unit
SD: Standard Deviation
SQ: Surprise Question
