# Supplementary material for: Prevalence and clinical characteristics of patients with Advanced Chronic Illness and Palliative Care needs, identified with the NECPAL CCOMS-ICO© Tool at a Tertiary Care Hospital
Source: BMC Palliat Care. 2022 Nov 28;21:210. doi: 10.1186/s12904-022-01101-4 (PMC9703744; doi:10.1186/s12904-022-01101-4)
Supplement: Supplementary file 1 — Supplementary Table S1. Distribution of admitted patients, those included, those identified with the Surprise Question and those identified with the NECPAL CCOMS-ICO © instrument for each service. [file 12904_2022_1101_MOESM1_ESM.pdf]

Supplemental Table S1. Distribution of admitted patients, those included, those identified with the Surprise Question and those identified with the NECPAL CCOMS-ICO © instrument for each service.

	Service	Inpatients n=602 n	Included n=236 n (%)*	PS + n=209 n (%)*	NECPAL+ n=202 n (%)*	NECPAL I-II n=105 n (%)*	NECPAL III n=97 n (%)*
1	Internal Medicine and GAU	46	19 (41.30%)	18 (39.13%)	16 (37.78%)	8 (17.39%)	8 (17.39%)
2	OS and Traumatology	61	19 (31.14%)	18 (29.50%)	17 (27.86%)	13 (21.31%)	4 (6.55%)
3	PCU	17	17 (100%)	17 (100%)	17 (100%)	2 (11.76%)	15 (88.23%)
4	Nephrology	20	15 (75%)	6 (30%)	6 (30%)	3 (15%)	3 (15%)
5	Neurology	38	15 (39.47%)	10 (26.31%)	10 (26.31%)	9 (23.68%)	1 (2.63%)
6	General and Digestive Surgery	52	15 (28.84%)	14 (26.92%)	14 (26.92%)	7 (13.46%)	7 (13.46%)
7	Medical Oncology	22	13 (59.09%)	12 (54.54%)	12 (54.54%)	5 (22.72%)	7 (31.81%)
8	EDSSU	24	13 (54.16%)	13 (54.16%)	13 (54.16%)	3 (12.50%)	10 (41.66%)
9	Clinical Hematology	28	13 (46.42%)	13 (46.42%)	13 (46.42%)	9 (32.14%)	4 (14.28%)
10	Cardiology & Cardiac Surgery	48	13 (27.08%)	13 (27.08%)	13 (27.08%)	5 (10.41%)	8 (16.66%)
11	Angiology and Vascular Surgery	22	12 (54.54%)	104 (5.45%)	10 (45.45%)	5 (22.72%)	5 (22.72%)
12	Psychiatry	23	12 (52.17%)	9 (39.13%)	9 (39.13%)	6 (26.08)	3 (13.04%)
13	Intensive Medicine	30	12 (40%)	11 (36.66%)	11 (36.66%)	9 (30%)	2 (6.66%)
14	Pneumology	30	11 (36.66%)	11 (36.66%)	11 (36.66%)	8 (26.66%)	3 (10%)
15	Neurosurgery	30	11 (36.66%)	10 (33.33%)	8 (26.66%)	4 (13.33%)	4 (13.33%)

	Service	Inpatients n=602 n	Included n=236 n (%)*	PS + n=209 n (%)*	NECPAL+ n=202 n (%)*	NECPAL I-II n=105 n (%)*	NECPAL III n=97 n (%)*
16	Digestive System	12	10 (83.33%)	10 (83.33%)	9 (75%)	4 (33.33%)	5 (41.66%)
17	Infectious Diseases	20	7 (35%)	6 (30%)	6 (30%)	0 (0%)	6 (30%)
18	Urology	23	3 (13.04%)	3 (13.04%)	3 (13.04%)	2 (8.69%)	1 (4.34%)
19	Maxillofacial Surgery	4	2 (50%)	2 (50%)	2 (50%)	2 (50%)	0 (0%)
20	Rheumatology	2	1 (50%)	1 (50%)	1 (50%)	0 (0%)	1 (50%)
21	Endocrinology and Nutrition	3	1 (33.33%)	0 (0%)	0 (0%)	0 (0%)	0 (0%)
22	Ophthalmology	3	1 (33.33%)	1 (33.33%)	0 (0%)	0 (0%)	0 (0%)
23	Plastic and Repair Surgery	20	1 (5%)	1 (5%)	1 (5%)	0 (0%)	1 (5%)
24	Thoracic Surgery	7	0 (0%)	-	-	-	-
25	Gynecology	6	0 (0%)	-	-	-	-
26	Otorhinolaryngology	11	0 (0%)	-	-	-	
27	Dermatology	0	-	-	-		
	**Total**	**602**	**236 (39.20%)****	**209 (34.71%)****	**202 (33.55%)****	**105** (17.44%)**	**97** (16.11%)**

* (%): The Percentages Of Both The Patients Included, As Well As The Surprise Question + Patients, The Necpal + Patients, The Necpal I-Ii And The Necpal Iii Of Each Service Are On The Total Number Of Patients Admitted To Each Service.

** Percentages With Respect To The Total Number Of Patients Admitted To The 2 Hospitals (602).

Abbreviations: %, Percentage; EDSSU, Emergency Department Short Stay Unit; GAU, Geriatric Assessment Unit; N, Number; OS, Orthopedic Surgery; PCU, Palliative Care Unit.
