# Supplementary material for: Prevalence and clinical characteristics of patients with Advanced Chronic Illness and Palliative Care needs, identified with the NECPAL CCOMS-ICO© Tool at a Tertiary Care Hospital
Source: BMC Palliat Care. 2022 Nov 28;21:210. doi: 10.1186/s12904-022-01101-4 (PMC9703744; doi:10.1186/s12904-022-01101-4)
Supplement: Supplementary file 2 — Supplementary Table S2. Prevalence and intensity of symptoms according to the ESAS scale. [file 12904_2022_1101_MOESM2_ESM.pdf]

Supplemental Table S2. Prevalence and intensity of symptoms according to the ESAS scale

Symptoms	Non NECPAL n= 14	NECPAL I-II n= 36	NECPAL III n= 29	TOTAL n=79	P value
**Pain**, n (%)					
≥ 1	11 (78.57%)	24 (66.66%)	19 (65.51%)	54 (68.35%)	
≥ 4	8 (57.1%)	17 (47.2%)	15 (51.7%)	40 (50.6%)	
Mean (SD)	4.07 (3.12)	3.08 (2.92)	3.76 (3.49)	3.51 (3.16)	
Median (range)	4.50 (0-10)	3 (0-9)	4 (0-10)	4 (0-10)	ns
**Tiredness**, n (%)					
≥ 1	10 (71.42%)	31 (86.11%)	25 (86.20%)	66 (83.54%)	
≥ 4	10 (71.4%)	28 (80%)	23 (79.3%)	61 (78.2%)	
Mean (SD)	5.57 (4.21)	5.54 (2.95)	5.90 (3.42)	5.68 (3.34)	
Median (range)	6.5 (0-10)	5 (0-10)	6 (0-10)	5.5(0-10)	ns
**Nausea**, n (%)					
≥ 1	1 (7.14%)	6 (16.6%)	4 (13.79%)	11 (13.92%)	
≥ 4	1 (7.1%)	4 (11.1%)	1 (3.4%)	6 (7.6%)	
Mean (SD)	0.57 (2.13)	0.83 (2.31)	0.55 (1.78)	0.68 (2.07)	
Median (range)	0 (0-8)	0 (0-10)	0 (0-9)	0 (0-10)	ns
**Depression**, n (%)					
≥ 1	13 (92.86%)	29 (80.55%)	21 (72.41%)	63 (79.75%)	
≥ 4	8 (57.1%)	23 (63.9%)	19 (65.5%)	50 (63.3%)	
Mean (SD)	4.29 (2.81)	4.61 (3.42)	5.03 (3.84)	4.71 (3.45)	
Median (range)	4.5 (0-10)	5 (0-10)	5 (0-10)	5 (0-10)	ns
**Anxiety**, n (%)					
≥ 1	9 (75%)	25 (69.45%)	22 (75.87%)	56 (73.42%)	
≥ 4	9 (75%)	24 (66.7%)	21 (72.4%)	54 (70.1%)	
Mean (SD)	5.83 (3.78)	5 (3.89)	5.66 (3.85)	5.38 (3.82)	
Median (range)	7 (0-10)	5 (0-10)	7 (0-10)	7 (0-10)	ns
**Drowsiness**, n (%)					
≥ 1	6 (42.86%)	20 (55.56%)	14 (48.28%)	40 (50.66%)	
≥ 4	6 (42.9%)	16 (45.7%)	10 (34.5%)	32 (41%)	
Mean (SD)	2.57 (3.29)	3.51 (3.76)	2.17 (2.8)	2.85 (3.36)	
Median (range)	0 (0-8)	2 (0-10)	0 (0-10)	1 (0-10)	ns
**Shortness of breath**, n (%)					
≥ 1	4 (28.58%)	14 (38.89%)	12 (41.38%)	30 (37.98%)	

Symptoms	Non NECPAL n= 14	NECPAL I-II n= 36	NECPAL III n= 29	TOTAL n=79	P value
≥ 4	3 (21.4%)	11 (30.6%)	12 (41.4%)	26 (32.9%)	
Mean (SD)	1.43 (2.71)	2.17 (3.01)	2.66 (3.34)	2.22 (3.11)	
Median (range)	0 (0-7)	0 (0-10)	0 (0-8)	0 (0-10)	ns
**Lack of appetite**, n (%)					
≥ 1	7 (50%)	22 (61.12%)	18 (62.07%)	47 (59.50%)	
≥ 4	7 (50%)	19 (52.8%)	14 (48.3)	40 (50.6%)	
Mean (SD)	3.79 (4.3)	4.19 (4.02)	4.34 (4.26)	4.18 (4.11)	
Median (range)	2 (0-10)	4.5 (0-10)	3 (0-10)	4 (0-10)	ns
**Insomnia**, n (%)					
≥ 1	12 (85.72%)	21 (58.34%)	13 (44.83%)	46 (58.23%)	
≥ 4	8 (57.1%)	14 (38.9%)	11 (37.9%)	33 (41.8%)	
Mean (SD)	5.14 (3.97)	3 (3.35)	3.52 (4.46)	3.57 (3.93)	
Median (range)	5 (0-10)	2 (0-10)	0 (0-10)	2 (0-10)	ns
**Perception of wellbeing**, n (%)					
≥ 1	11 (78.58%)	28 (77.78%)	22 (75.87%)	61 (77.22%)	ns
≥ 4	8 (57.1%)	26 (72.2%)	22 (75.9%)	56 (70.9%)	
Mean (SD)	3.71 (2.46)	5.25 (3.56)	5.69 (3.84)	5.14 (3.53)	
Median (range)	5 (0-8)	5 (0-10)	5 (0-10)	5 (0-10)	ns

Abbreviations: n, number of patients; %: percentage of patients; SD, standard deviation; ns, differences not statistically significant
